# The effect of Ganoderma lucidum extract on immunological function and identify its anti-tumor immunostimulatory activity based on the biological network

**DOI:** 10.1038/s41598-018-30881-0

**Published:** 2018-08-23

**Authors:** Ruolin Zhao, Qilong Chen, Yu-min He

**Affiliations:** 1 Institute of Interdisciplinary Medicine Research, Shanghai University of Traditional Chinese Medicine, Shanghai, 201203 China; 2 Research Center for TCM Complexity System, Shanghai University of Traditional Chinese Medicine, Shanghai, 201203 China

## Abstract

*Ganoderma lucidum* extract (GLE) has shown positive effects for tumor treatment. However, the molecular mechanism of GLE treatment is unknown. In this study, a Hepa1-6-bearing C57 BL/6 mouse model was established to explore the anti-tumor and immunostimulatory activity of GLE treatment. The results showed that GLE effectively inhibited tumor growth without hepatic/renal toxicity and bone marrow suppression, and might enhancing immunological function. Based on the mRNA profiles of GLE treated and untreated mice, 302 differentially expressed (DE) mRNAs were identified and 6 kernel mRNAs were identified from the established protein-protein interaction (PPI) network. Quantitative RT-PCR and western-blot analysis indicated that 6 mRNAs have had statistically significant differences between the GLE treated and untreated mice. Furthermore, four kernel pathways were isolated from the KEGG-Target network, including the Jak-STAT signaling pathway, T cell receptor signaling pathway, PI3K-Akt signaling pathway, and cytokine-cytokine receptor interaction. Western-blot and cytokine detection results demonstrated that GLE suppressed growth and proliferation of tumors by the Jak-STAT signaling pathway, T cell receptor signaling pathway and PI3K-Akt signaling pathway, but also regulated the expression levels of serum immune cytokines and improved the anti-tumor immunostimulatory activity.

## Introduction

Cancer, the second leading cause of death worldwide, is a major global public health problem^1^. At present, chemotherapy is mainly recommended for patients with extrapelvic metastases or recurrent disease who are not candidates for radiotherapy or exenterative surgery^2^. However, drug resistance and dose-limiting toxicities are the major problems leading to termination or failure of successful treatment in the clinic^3^. In particular, chemotherapy can reduced or damaged immune function and negated the therapeutic benefits^4^. Thus, novel anti-cancer drugs are required to kill cancer cells effectively as well as alleviate toxicity.

The immune system plays a vital role in tumor treatment. Immune-targeted therapies have demonstrated durable responses and prolong survival time for many tumor types, especially in cases with limited treatment options and poor overall prognosis^5^. Important immune organs such as spleen and thymus as well as immune cells including macrophages, lymphocytes, natural killer (NK) cells, play important roles in anti-cancer effect. In addition, cytokines produced by the inflammatory cells in the tumor microenvironment are closely associated with cancer cell survival, proliferation, differentiation and progression^6^.

*Ganoderma lucidum* (GL), a traditional medicinal fungus, has been widely used as an adjuvant in clinical anti-tumor therapies^7,8^. *Ganoderma lucidum* polysaccharides (GLPS) and triterpenoids are the main constituents of *Ganoderma lucidum*^9^, which have anti-tumor activating properties on macrophages, T lymphocytes, natural killer (NK) cells, dendritic cells, as well as initiating the production of cytokines^10–15^. The studies demonstrate that GLPS significantly suppresses tumorigenesis, invasion and metastasis in several cancers^16^, especially, it can protect the liver with little toxicity as well as reduce toxicity-related conditions due to chemotherapy on normal cells^10,11,17^. The triterpenes of GL can suppress proliferation, induce cell cycle arrest and apoptosis, inhibit angiogenesis and invasion in cancer^18^, but also can improve immunity of tumor patients by affecting immune-related signal transduction pathways^19^. In previous work, we extracted the GLPS and triterpenoids from [*Ganoderma lucidum* (Leyss. Ex Fr.) Karst.] and [*Ganoderma sinense* Zhao, Xu et Zhang], and named *Ganoderma lucidum* extract (GLE) complex. The primarily study reveal that the GLE can prolong the survival rate of cancer patients while also improving the patient’s quality of life^20^. However, the anti-tumor and immunological effects of GLE in the tumor treatment process remain unclear.

In this study, we aimed to explore the anti-tumor and immunostimulatory activity of GLE in tumor treated process. Firstly, we established the Hepa1-6-bearing mouse model in C57 BL/6 mice, and demonstrated that the GLE might enhance immunological function and effectively inhibited tumor growth *in vivo* without hepatic/renal toxicity and bone marrow suppression. Secondly, the mRNA profiles of GLE treated and untreated Hepa1-6-bearing mice were detected and 302 differential expressed (DE) mRNAs were identified. Based on the protein-protein interaction (PPI) network and KEGG-Target network, 6 kernel mRNAs and 4 immune related pathways were screened in GLE treatment process. The qRT-PCR and Western-blot demonstrated that these 6 kernel mRNAs significantly improved the efficient diagnostic accuracy of GLE. Western-blot and serum cytokine detection suggested that 4 pathways were highly correlated with GLE treatment process. This study indicated that GLE may have potential immuno-modulating effect in the tumor treatment process.

## Results

### GLE suppressed tumor growth

**Figure 1:**
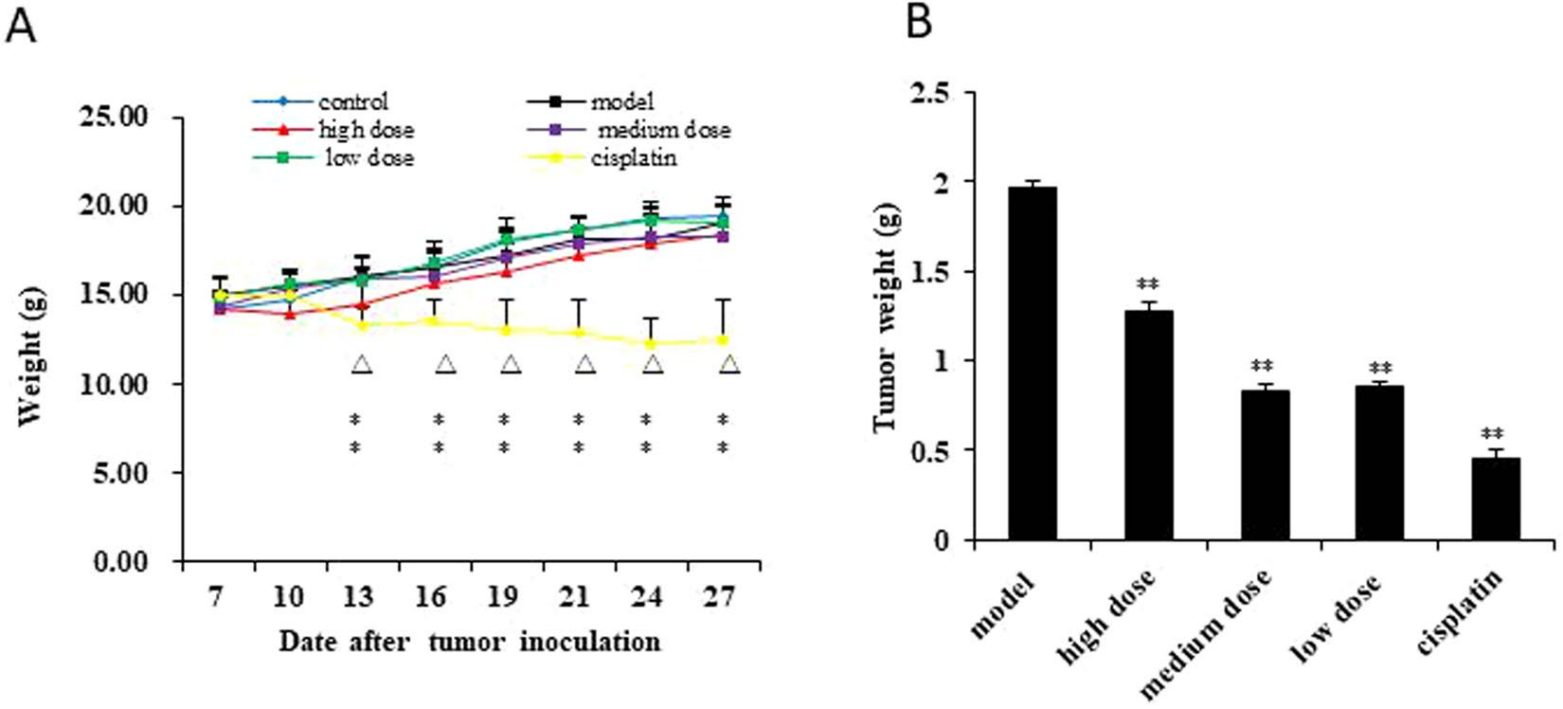
GLE effectively inhibited tumor growth *in vivo*. (**A**) The weight of mice was significantly reduced in cisplatin group from the 10th day (*P* < 0.01) compared with those in model group and control group. The difference was no statistically significant in GLE treatment group. (**B**) The average tumor weight in GLE treatment and cisplatin groups decreased significantly compared with those in model group (*P* < 0.01). Representative results of three independent experiments are shown. Error bars, SD; ^*^P < 0.05; ^**^P < 0.01, versus control values; ^Δ^*P < *0.05; ^ΔΔ^*P* < 0.01, versus model values.

To evaluate the anti-cancer activity of GLE *in vivo*, we established a Hepa1-6-bearing mice model. The average body weights show that the cisplatin group was significantly reduced from the 10th day (*P* < 0.01), however, the GLE treatment exhibited few effects on the body weights for Hepa1-6-bearing mice (Fig. 1A). After a 28-day treatment, the transplanted tumors of mice were dissected out and immediately weighted. The results show that the average tumor weight in GLE and cisplatin groups were decreased significantly (Fig. 1B). Furthermore, the inhibit rate of tumor of cisplatin group was highest than other groups, but the average body weight of this group also was dramatically decreased. Body weight can reflect growth and physical condition of mice, this phenomenon suggested that GLE can inhibit tumor growth, but also improve the quality of life.

### The toxicological impacts of GLE on Hepa1-6-bearing mice

**Figure 2:**
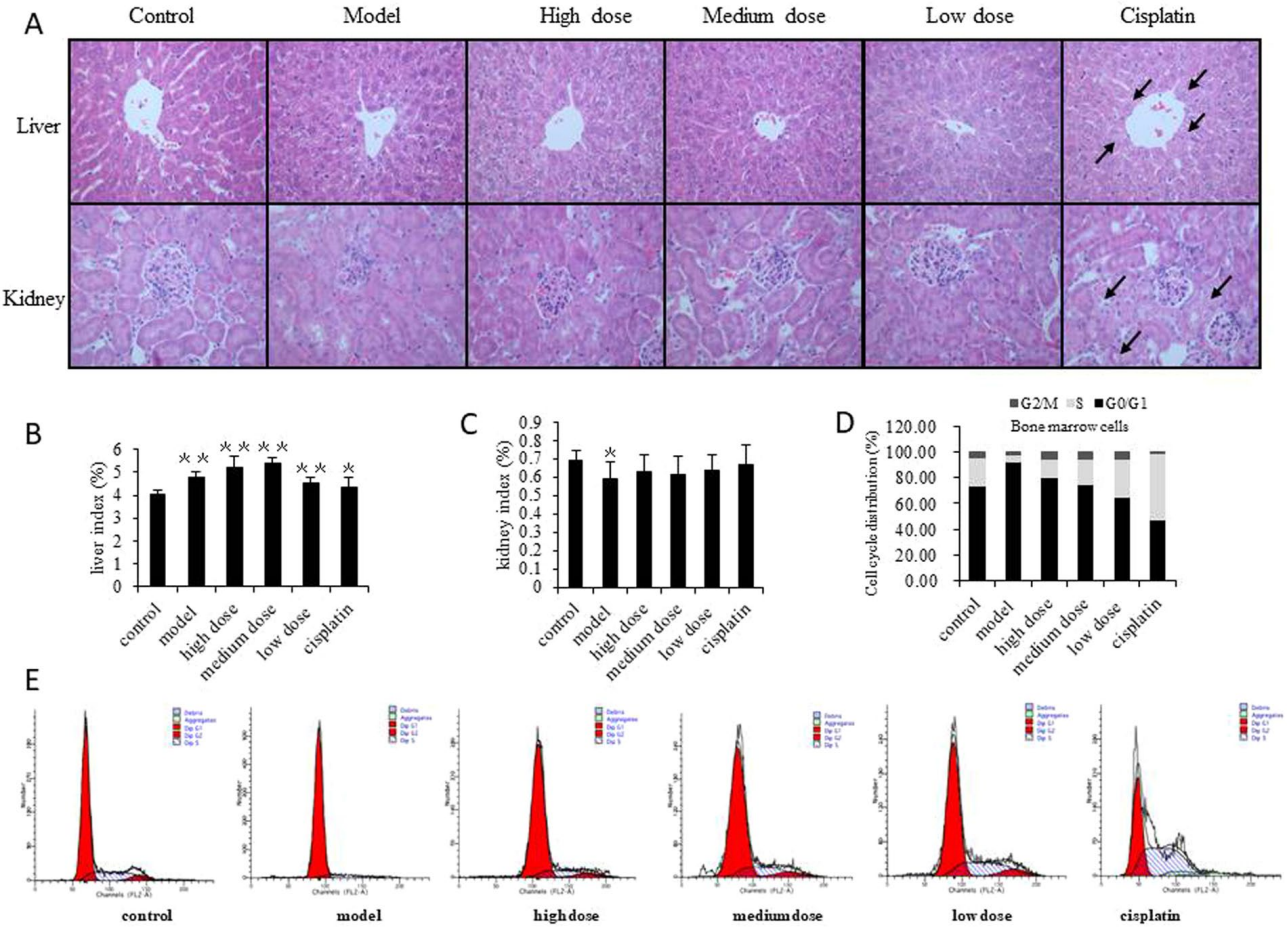
The toxicological impact of GLE treated tumor-bearing mice. (**A**) Pathological examination was used to detect toxic damage of liver and renal tissue in all groups. Sections of liver and renal tissue in mice were stained using HE followed by observation on a phase-contrast microscope (×400). Obvious changes are highlighted by arrows. The structure of the hepatic lobule was intact, and no inflammatory cell infiltration was found in the portal area and the liver proper. Six hours prior to sacrifice, mice had not been fasted, thus each group showed vacuoles in the liver cells. This simply indicated glycogen accumulation in the liver cells was not associated with pathology. Likewise, hypertrophy of hepatocytes around the central vein of the liver tissue in cisplatin group was attributed to damage to liver cells caused by cisplatin. (**B**,**C**) Effects of GLE on liver and renal indexes were analyzed in tumor-bearing mice. (**D**,**E**) GLE improves the suppression of bone marrow cells in tumor-bearing mice. FACS was used to detect the effects of GLE on the cell cycle progression of bone marrow. Representative results of three independent experiments are shown. Error bars, SD; ^*^P < 0.05; ^**^P < 0.01, versus control values; ^Δ^*P* < 0.05; ^ΔΔ^*P* < 0.01, versus model values.

The HE staining results showed that the liver tissue of the model and the GLE group had no obvious abnormalities compared with normal liver tissue (Fig. 2A). However, the liver indexes show that the GLE, model and cisplatin groups were slightly larger than the control group (Fig. 2B). The kidney indexes show the model group was significantly decreased (*P* < 0.05), while the other groups have no statistical difference each other (Fig. 2C). Specifically, there was no cell proliferation in the glomeruli or renal tubular epithelial cells which were found intact. In addition, no tubular type was found in the lumen (Fig. 2A). Renal tissue in the cisplatin group showed prominent proximal tubular epithelial cell swelling. Within renal tubules, cytoplasmic visible particles and vacuolar degeneration were also observed caused by cisplatin toxicity (Fig. 2A).

To determine the toxic effects on normal hepatocytes and nephrocytes, the serum hepatic and renal function markers were analyzed in all experimental groups. In cisplatin group, the serum activities of ALT and AST increased l.68- and 2.21-fold (Table 1), and serum Scr and BUN increased 1.26- and 1.95-fold (Table 2), respectively. However, these parameters remained largely unchanged in all of the GLE treated groups. It reveals that there are very low hepatic and renal toxicity in GLE treated tumor-bearing mice.

**Table 1:** Effects of GLE on serum hepatic function in tumor-bearing mice.

Indexes	Control	Model	High dose	Medium dose	Low dose	Cisplatin
ALT(U/L)	30.00 ± 3.08	41.00 ± 8.69^*^	45.00 ± 2.74^ΔΔ^	34.40 ± 2.07^ΔΔ^	33.00 ± 3.74	50.40 ± 6.95^**^
AST(U/L)	119.00 ± 21.30	254.00 ± 39.12^**^	166.80 ± 11.82^ΔΔ^	149.00 ± 27.45^ΔΔ^	164.00 ± 10.15^ΔΔ^	262.80 ± 21.50^**^
TBIL(μmol/L)	2.20 ± 0.45	3.50 ± 0.50^**^	2.40 ± 0.55	1.80 ± 0.84	1.60 ± 0.55^ΔΔ^	2.40 ± 0.89

Note: ^*^P < 0.05; ^**^P < 0.01, versus control values; ^Δ^*P* < 0.05; ^ΔΔ^*P* < 0.01, versus model values.

**Table 2:** Effect of GLE on renal function in tumor-bearing mice.

Indexes	Control	Model	High dose	Medium dose	Low dose	Cisplatin
Scr(μmol/L)	14.60 ± 2.30	14.60 ± 1.52	13.60 ± 0.89	14.40 ± 1.14	13.20 ± 1.48	18.40 ± 1.52^*,ΔΔ^
BUN(mmol/L)	8.64 ± 2.48	11.72 ± 1.77	11.22 ± 1.62	11.14 ± 1.22	11.88 ± 2.19	16.84 ± 2.57^**,Δ^

Note: ^*^P < 0.05; ^**^P < 0.01, versus control values; ^Δ^*P* < 0.05; ^ΔΔ^*P* < 0.01, versus model values.

Furthermore, the cell cycle progression of bone marrow was detected by flow cytometry. The results showed that the bone marrow cells of model group were triggered G0/G1 phase arrest, the cisplatin group was triggered G2/M phase arrest, while the GLE reduced the percentage of bone marrow cell distribution of G0/G1 phase transforming to G2/M and S phase. (Fig. 2D,E). It means that the cisplatin could induce bone marrow suppression while GLE decrease bone marrow arrest. This phenomenon demonstrated that the GLE presented undetectable toxic risk for the vital organs in tumor-bearing mice and had no bone marrow suppression.

### The effect of GLE on the immune system

**Figure 3:**
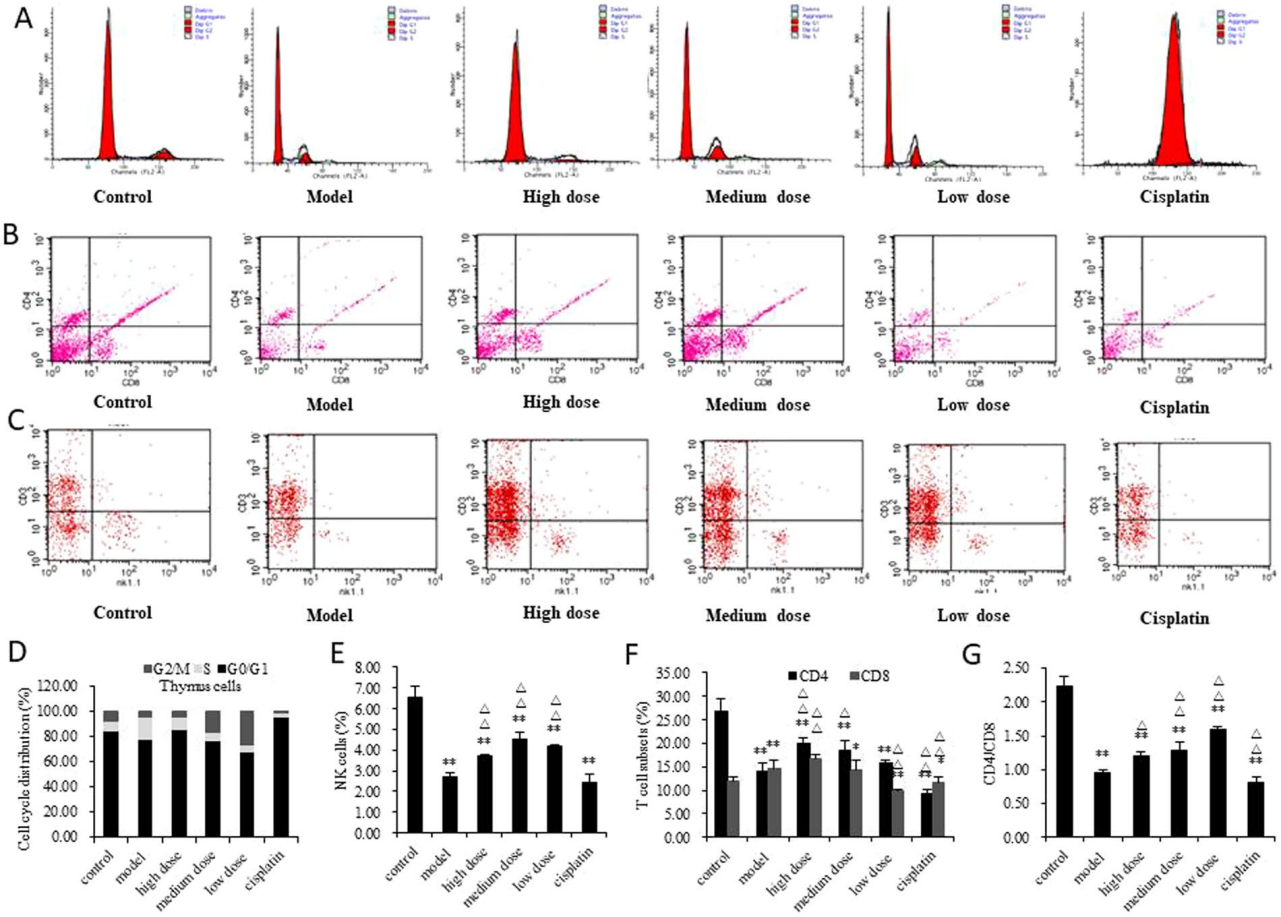
Effect of GLE on immunostimulatory activity of tumor-bearing mice was determined. (**A**,**D**) GLE improved the suppression of thymus cells in tumor-bearing mice. Cell cycle distribution was analyzed by FACS, in order to detect the effect of GLE on the cell cycle progression of thymus. (**B**,**F**,**G**) Effects of GLE on T cell subsets in peripheral blood in tumor-bearing mice were detected. The lymphocyte subsets were detected by flow cytometry. The lymphocyte subset analysis included CD3+ (T lymphocyte), CD4+ (T-helper cells) and CD8+ (T-suppressor cells). Representative results of three independent experiments are shown. (**C**,**E**) Effects of GLE on NK cell activity in tumor-bearing mice. The percentage of NK cells were quantified by FACS. Error bars, SD; ^*^P < 0.05; ^**^P < 0.01, versus control values; ^Δ^*P* < 0.05; ^ΔΔ^*P* < 0.01, versus model values.

Thymus is an important immune organ in the body. As show in Fig. 3, the thymocytes of the model group produced G2/M and S phase arrest and the cisplatin group produced G0/G1 phase cell cycle arrest, however, GLE decreased thymocyte distributions in G2/M phase arrest and transformed to S phase (Fig. 3A,D). In cisplatin group, concentrations of WBC and LY show that the amount of WBC and LY was dramatically decline, however, GLE could elevate the amount of WBC and LY in the blood (Table 3). The activities of NK cells were determined, and the results show that the numbers of NK cells were all significantly decreased in model, GLE and cisplatin groups (Fig. 3C,E), but the decreasing rate of NK cells of GLE treatment was less than model and cisplatin group (Fig. 3E). It was indicated that GLE effectively increased the number of NK cells in tumor-bearing mice. The lymphocyte subset analysis shows that the CD4+ and CD4+/CD8+ T cells of model and cisplatin groups were decreased significantly, but GLE group was dramatically elevated (Fig. 3B,F,G).

**Table 3:** Effect of GLE on blood WBC and LY of tumor-bearing mice.

Concentration	Control	Model	High dose	Medium dose	Low dose	Cisplatin
WBC(10^9^/L)	3.80 ± 0.32	3.93 ± 0.06	4.85 ± 0.39^*Δ^	4.65 ± 0.60^*^	4.73 ± 0.06^*,ΔΔ^	1.60 ± 0.36^**,ΔΔ^
LY(%)	77.40 ± 5.13	65.80 ± 1.57^*^	75.13 ± 2.05^ΔΔ^	65.18 ± 3.40	73.20 ± 6.17^Δ^	48.80 ± 3.56^**,ΔΔ^

Note: ^*^P < 0.05; ^**^P < 0.01, versus control values; ^Δ^*P* < 0.05; ^ΔΔ^*P* < 0.01, versus model values.

These results indicated that cisplatin had a strong toxic impact for the immune system, but GLE stimulated an anti-tumor immune response in tumor-bearing mice treatment process. Importantly, this stimulation of an antitumor immune response might contribute to the antitumor activity of GLE *in vivo*.

### Differentially expressed mRNAs identification

In this work, the mRNA profiles of 6 cases GLE treated and untreated Hepa1-6-bearing C57 BL/6 mice were detected by microarray. The efficacy of medium dose GLE was best among the high, medium and low dose, thus, only medium dose GLE treat Hepa1-6-bearing C57 BL/6 mice was selected for microarray detection. The microarray data were deposited in Gene Expression Omnibus (GEO) database, and the accession number is GSE117503.

**Figure 4:**
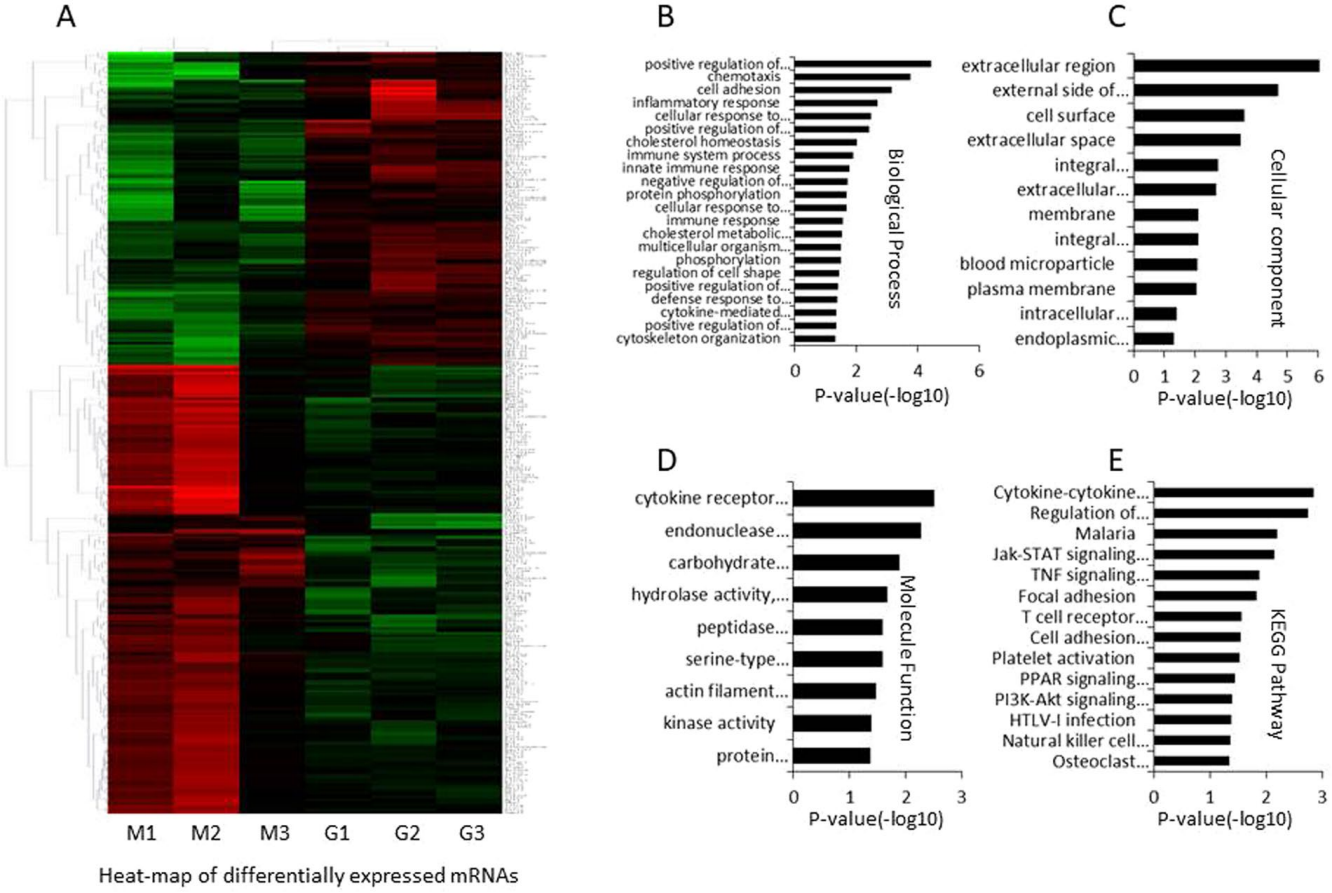
The differential expressed mRNAs were identified between the GLE treated and untreated Hepa1-6-bearing C57 BL/6 mice. (**A**) Heat-map of DE mRNAs between GLE treated and untreated Hepa1-6-bearing C57 BL/6 mice. The red color represents up-expression of mRNA and green color represents down-expression of mRNA. The relationship among the samples was divided by binary tree classification and was shown at the upper portion. Hierarchical cluster of mRNAs were displayed at nearside. (**B**–**D**) GO (Gene Ontology) terms in differentially expressed mRNAs. (**E**) KEGG pathways in differentially expressed mRNAs.

Using the R package, 302 differentially expressed (DE) mRNAs were identified based on fold change >2 and *P* < 0.05, including 178 upregulated mRNAs and 124 downregulated mRNAs (Fig. 4a). The results suggested that the levels of mRNAs are difference between the GLE group and model group.

### Enrichment analysis of differentially expressed mRNAs

To understand the DE mRNAs holistically, we conducted functional enrichment analysis using DAVID online. Gene ontology (GO) analysis shows that the Biological Process (BP) were associated with positive regulation of cholesterol efflux, chemotaxis, cell adhesion, inflammatory response, cellular response to tumor necrosis factor, positive regulation of cAMP biosynthetic process, cholesterol homeostasis, immune system process, innate immune response, negative regulation of peptidase activity, protein phosphorylation, cellular response to interleukin-1, immune response, cholesterol metabolic process, multicellular organism development, phosphorylation, regulation of cell shape, positive regulation of protein kinase B signaling, defense response to bacterium, cytokine-mediated signaling pathway, positive regulation of cytosolic calcium ion concentration, and cytoskeleton organization (Fig. 4B). The cellular component (CC) terms were associated with extracellular region, external side of plasma membrane, cell surface, extracellular space, integral component of plasma membrane, extracellular exosome, membrane, integral component of endoplasmic reticulum membrane, blood microparticle, plasma membrane, intracellular membrane-bounded organelle, and endoplasmic reticulum lumen (Fig. 4C). Furthermore, the cytokine receptor activity, endonuclease activity, carbohydrate binding, hydrolase activity, acting on glycosyl bonds, peptidase inhibitor activity, serine-type endopeptidase inhibitor activity, actin filament binding, kinase activity, and protein homodimerization activity were highly correlated with Molecule Function (MF) (Fig. 4D). These results suggested that the immune related GO terms more important than others in GLE treatment.

KEGG pathways showed that the cytokine-cytokine receptor interaction, Jak-STAT signaling pathway, T cell receptor signaling pathway, TNF signaling pathway, PPAR signaling pathway, PI3K-Akt signaling pathway, cell adhesion molecules (CAMs), regulation of lipolysis in adipocytes, malaria, platelet activation, HTLV-I infection, NK cell mediated cytotoxicity, Osteoclast differentiation, and Focal adhesion were highly associated with the DE mRNAs. Interestingly, the signal-related pathways had an impressive function for differentially expressed mRNAs, and suggesting that these pathways acted as critical roles in GLE treatment process (Fig. 4E).

### Protein-protein interaction (PPI) network

**Figure 5:**
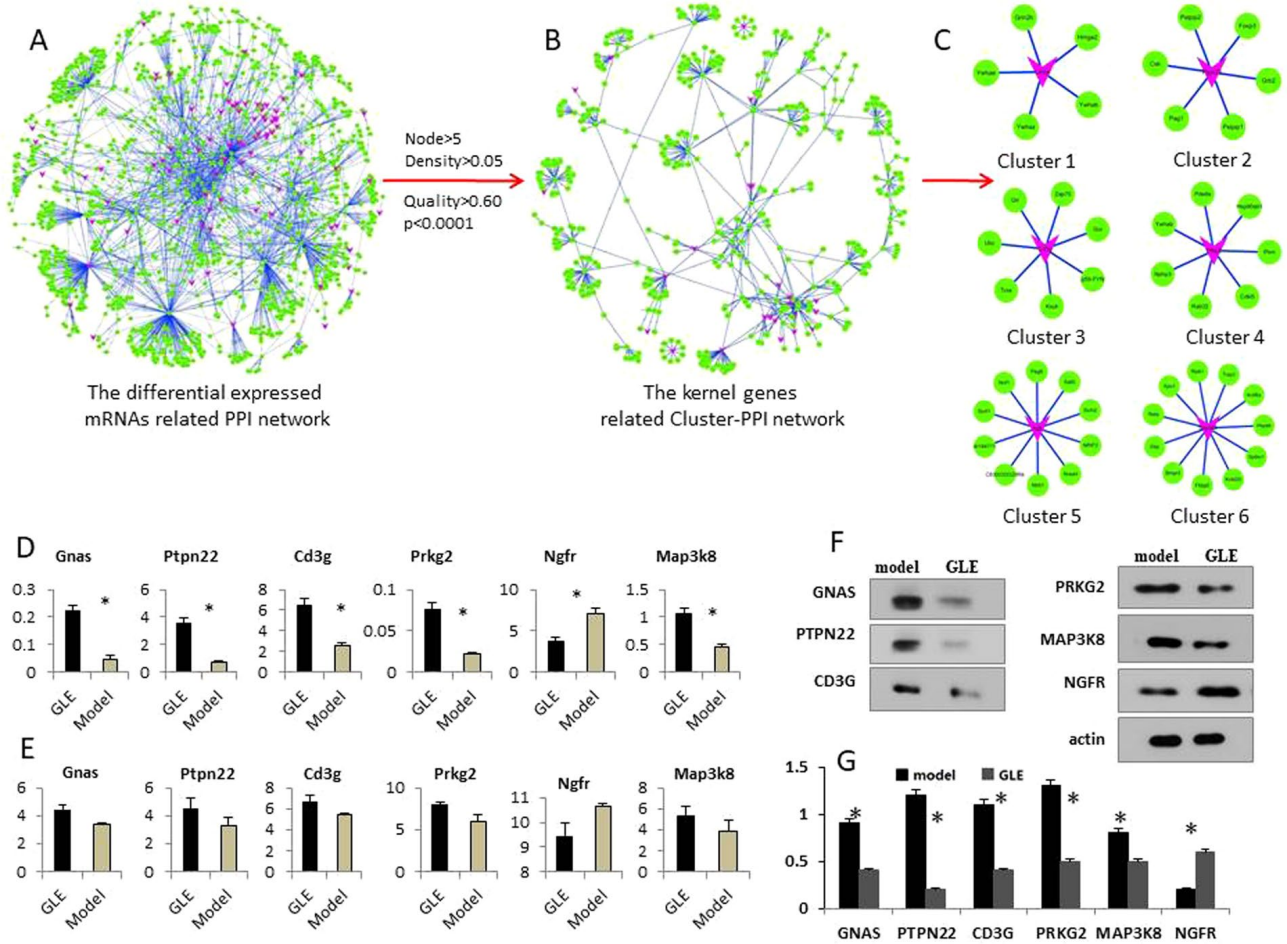
The protein-protein interaction (PPI) network was constructed based on DE mRNAs, the related clusters were identified using ClusterONE algorithm. (**A**) The global profiles of GLE treatment related PPI network was built. The red nodes represent the differential expressed mRNAs. (**B**) The topological profile of GLE treatment related Cluster-PPI network, the red nodes represent the differential expressed mRNAs. (**C**) The kernel mRNA clusters isolated from Cluster-PPI network. (**D**) The qRT-PCR was used to validate 6 potential kernel mRNAs, and the expression levels of 6 kernel mRNAs have statistically significant difference in GLE treated Hepa1-6-bearing C57 BL/6 mice. (**E**) The expression tendency of 6 kernel mRNAs in the microarray. (**F**,**G**) Western-blot assay was used to further validate the 6 kernel mRNAs. Representative results of three independent experiments are shown. Error bars, ^*^P < 0.01 versus model values. Representative results of three independent experiments are shown. The β-actin was used as a loading control. Error bars, ^*^P < 0.01 versus model values.

Using the differentially expressed (DE) mRNAs and related pairs, the protein-protein interaction (PPI) network of GLE treatment was constructed. To investigate the biological functions of differentially expressed mRNAs in the network, we evaluated the intersections of the PPI pairs and the DE mRNAs. The topological profile shows that the network consists of a regulatory core structure, and related kernel nodes represent the most prominent functions (Fig. 5A). Importantly, these kernel nodes might act as determinants in the realized network profiles^21^. Such being the case, 16 clusters were isolated from the PPI network using ClusterONE algorithm (*P*-value < 0.001, node size > 6, and network density >0.05). Furthermore, the Cluster-PPI network was reconstructed by 16 isolated clusters (Fig. 5B), and the topological profile revealed that the correlation of clusters is stringently tight, suggesting that these clusters might play prominently functions in GLE treatment process.

### Kernel mRNAs screening and validating

The mRNA clusters might play important roles in GLE treatment progression and act as potential biomarkers for efficient evaluation of GLE. Such The parameters of Cluster-PPI network were calculated, and the kernel nodes were defined as BC ≥ Avg (BC), CC ≥ Avg (CC) and De ≥ Avg (De). Finally, 6 kernel mRNAs were screened from the Cluster-PPI network, including CD3G, GNAS, MAP3K8, NGFR, PRKG2, and PTPN22.

To measure the expression levels of mRNAs, the qRT-PCR was performed, and the results showed that these 6 kernel mRNA had statistical significance in GLE-treated Hepa1-6-bearing C57 BL/6 mice (Fig. 4D). Interestingly, the expression tendencies of these kernel mRNAs were coherent between the qRT-PCR and microarray (Fig. 4E). The Western-blot demonstrated that the expression levels of CD3G, GNAS, MAP3K8, NGFR, PRKG2 and PTPN22 were all down-regulated, which was consistent with qRT-PCR data (Fig. 5F,G) (Supplementary information, data 1). These results indicated that the levels of 6 kernel mRNAs might be beneficial to evaluating the efficacy of GLE.

### Kernel KEGG pathways screening and validating

**Figure 6:**
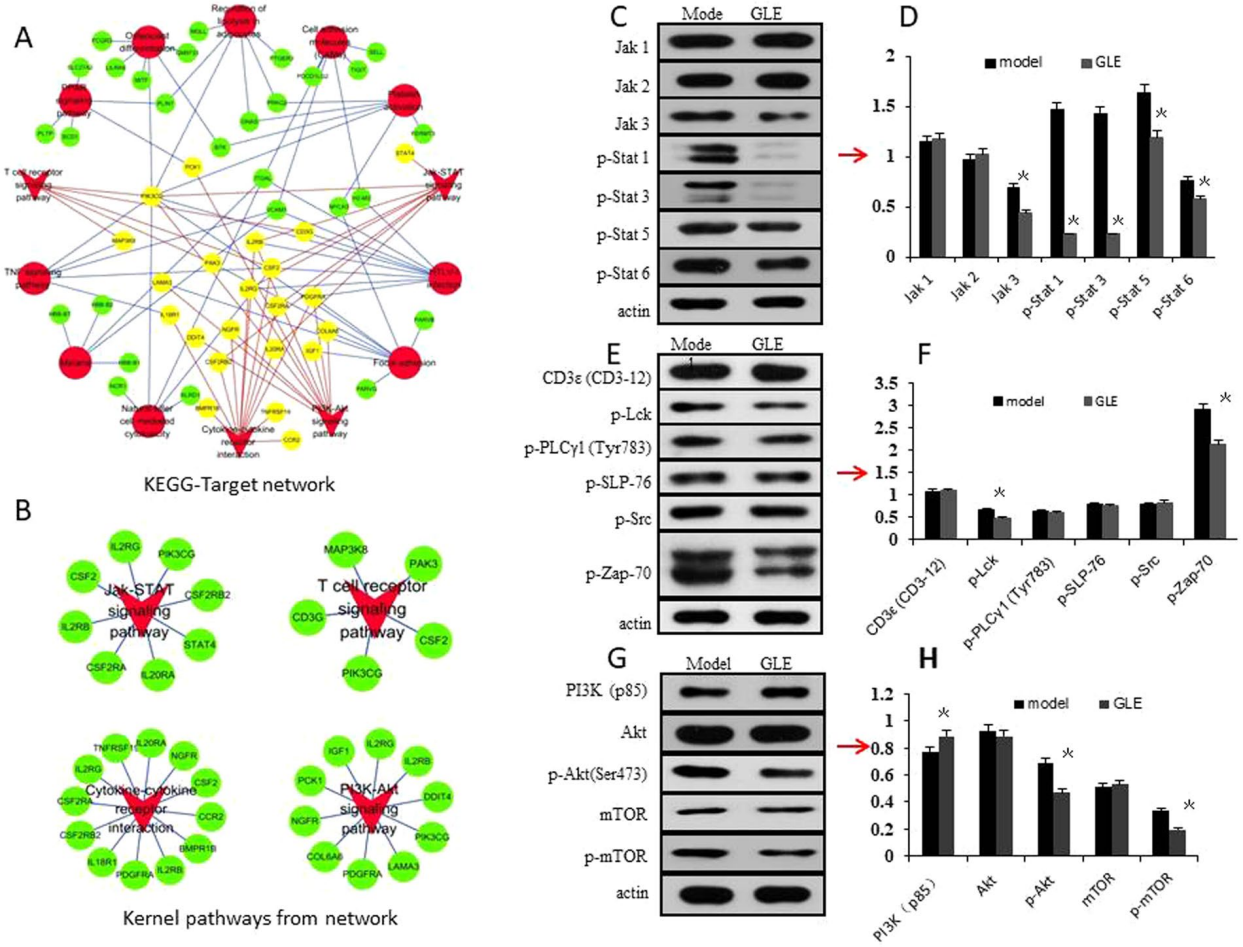
The kernel biological pathway screening and validating was based on the KEGG-Target network and Western-blot. (**A**) The KEGG-Target pathway network was constructed based on the differential expressed genes and their KEGG pathways. The red dot represents each KEGG pathway, the green dot represents target genes, and the red triangles represent the selected important pathways in network, including Jak-STAT signaling pathway, T cell receptor signaling pathway, PI3K-Akt signaling pathway, and cytokine-cytokine receptor interaction. The yellow dot represents the target genes of four selected pathways. (**B**) Four kernel pathways selected from the KEGG-Target network. (**C**,D) The effects of GLE were validated on the Jak/Stat signaling pathway. Compared with those in the model group, the expression levels of JAK3, p-Stat1, p-Stat3, p-Stat5 and p-Stat6 were significantly inhibited by GLE *in vivo* (*P* < 0.05). (**E**,**F**) The effects of GLE were validated on the T cell receptor signaling pathway. The expression levels of p-Lck and p-Zap-70 were down-regulated (*P* < 0.05). (**G**,**H**) The effects of GLE were validated on the PI3K/Akt/mTOR signaling pathway for proliferation inhibition. Compared with those in model group, the expression levels of p-Akt and p-mTOR were down-regulated (*P* < 0.05), and PI3K was up-regulated (*P* < 0.05). Densitometry analysis of the levels of these proteins relative to actin was performed. Representative results of three independent experiments are shown. The β-actin was used as a loading control. Error bars, SD; ^*^P < 0.05, versus model values.

To screen the kernel pathways of the DE mRNAs, the KEGG-Target network was constructed using KEGG pathways and target genes (Fig. 6A). The topological profile shows that the Jak-STAT signaling pathway, T cell receptor signaling pathway, PI3K-Akt signaling pathway, and cytokine-cytokine receptor interactionplay important roles in the KEGG-Target network (Fig. 6B).

Generally, the Jak/Stat signaling pathway, T cell receptor signaling pathway and PI3K/Akt/mTOR signaling pathway play important roles in moderation of anti-tumor immune defense. Western-blot showed that the expression levels of JAK3, p-Stat1, p-Stat3, p-Stat5, p-Stat6, p-Lck, p-Zap-70, p-Akt and p-mTOR were downregulated, and PI3K was upregulated (*P* < 0.05) (Fig. 6C–H) (Supplementary information, data 2). These results revealed that GLE might regulate immune function through suppressing Jak/Stat signaling pathway, T cell receptor signaling pathway and PI3K/Akt/mTOR signaling pathway.

Using serum cytokine protein chip, 20 kinds of cytokine concentration were detected between model and GLE groups, and 7 cytokines were significantly different in the serum of the GLE group, including IFN-γ, IL-4, IL-6, IL-9, IL-17, RANTES and TNF-α (Table 4). The array data were deposited in Protein Microarray Database (PMD), and the accession number is PMDA179. Importantly, the levels of IFN-γ, IL-1β, IL-4, IL-9, IL-12, RANTES and TNF-α were increased in the serum of the GLE group (Table 4). These results suggested that the regulation of serum immune cytokines were high correlated with the anti-tumor immunostimulatory activity of GLE.

**Table 4:** The changes of serum cytokine concentration in mice after GLE treatment (*x* ± *s*, n = 3).

Cytokine	Control	Model	GLE
GM-CSF	25.39 ± 1.74	22.89 ± 0.71	29.11 ± 5.04
IFN-γ	138.511 ± 5.00	116.72 ± 1.23^*^	184.65 ± 4.35^**,ΔΔ^
IL-1a	82.77 ± 5.30	74.31 ± 1.72	132.8 ± 41.64
IL-1b	143.02 ± 6.79	136.32 ± 39.36	176.27 ± 31.94^Δ^
IL-2	260.75 ± 13.80	161.26 ± 13.52^*^	333.71 ± 101.76
IL-3	5.00 ± 0.14	5.92 ± 2.57	6.31 ± 0.88
IL-4	1.87 ± 0.07	1.33 ± 0.43	6.10 ± 0.89^*,Δ^
IL-5	7.06 ± 0.46	16.01 ± 2.92^*^	13.75 ± 3.85
IL-6	21.17 ± 0.16	41.98 ± 8.56	42.28 ± 7.59*
IL-9	312.20 ± 0.34	111.04 ± 2.32^**^	390.26 ± 0.46^**,ΔΔ^
IL-10	271.02 ± 66.32	283.57 ± 38.61	314.09 ± 45.92
IL-12	105.30 ± 26.37	54.90 ± 3.48	110.72 ± 17.74^Δ^
IL-13	26.29 ± 0.02	44.61 ± 9.57	27.82 ± 2.21
IL-17	7.57 ± 0.29	8.82 ± 3.48	11.02 ± 0.86^*^
KC	0.54 ± 0.38	5.61 ± 2.17^*^	1.02 ± 0.31
MCP-1	589.63 ± 36.40	315.41 ± 46.02	613.42 ± 124.53
M-CSF	118.30 ± 25.68	127.09 ± 21.10^**^	123.21 ± 27.69
RANTES	3.58 ± 0.42	7.45 ± 0.33^**^	15.68 ± 1.77^*,Δ^
TNFa	851.19 ± 68.10	803.139 ± 36.36	1129.72 ± 85.82^Δ^
VEGF	33.65 ± 0.94	48.54 ± 6.44	33.69 ± 4.53

Note: Date in Table 4 was based on Log-Log Regression Standard Curves. ^*^P < 0.05; ^**^P < 0.01, versus control values;^Δ^*P < *0.05; ^ΔΔ^*P* < 0.01, versus model values.

## Discussion

In this study, we demonstrated that the *Ganoderma lucidum* extract (GLE) significantly inhibited the growth of tumors in Hepa1-6-bearing mice. The pathological and serum examination show that the GLE is safety and have not any adverse effects, but cisplatin have substantial hepatotoxicity and nephrotoxicity in tumor-bearing mice. Flow cytometry revealed that GLE reduced the percentage of bone marrow cell distribution in G0/G1 phase, transforming to G2/M and S phase (Fig. 2C). These results suggested that GLE presented undetectable toxic risk for vital organs and bone marrow suppression in tumor-bearing mice.

Furthermore, we found that GLE significantly potentiated immunomodulatory activity associated with an increase in CD4+ T cells and the ratio of CD4+/CD8+ T cells *in vivo*, and contributed significantly to antitumor activity (Fig. 3c). CD4^+^ T cells play a critical role in antitumor immune defense^22^, and CD8^+^ T cells are higher in stronger immune suppression. A disruption in CD4^+^/CD8^+^ balance lead to immune response disorder and leaving the body’s immune function in an immunosuppressive state^23,24^. In our study, GLE effectively elevated the number of NK cells in tumor-bearing mice, while the effect of cisplatin was the opposite (Fig. 3b). Generally, low NK activity was high correlated with the poor prognosis in advanced cancer^25^. To enhance NK activity, a number of immunotherapeutic approaches were used, such as IL-2 has been investigated in advanced stage cancer^26^. These results indicated that the GLE can stimulate anti-tumor immune response in the treated process of tumor-bearing mice.

Based on mRNA profiles, 302 DE mRNAs were identified from GLE treated and untreated Hepa1-6-bearing mice. Furthermore, 6 kernel mRNAs were screened from the established PPI network, including CD3G, GNAS, MAP3K8, NGFR, PRKG2, and PTPN22. The qRT-PCR and Western-blot demonstrated that these mRNAs have had statistically significant difference between the GLE treated and untreated tumor mice (Fig. 5C). The result suggested that these mRNAs might act as biomarkers to improve the diagnostic accuracy of efficient evaluation for GLE treatment. Interestingly, CD3G can be used as suitable biomarkers to distinguishing cancer in the very early stages of its development^27^. GNAS mutation occurred in gastric cancer^28^, and GNAS-mutated carcinoma was arising from gastric foveolar metaplasia in the duodenum after 9 years of observation^29^. MAP3K8 is a member of the serine/threonine protein kinase family^30^, which considered as a tumor-promoting oncogene in tumor^31^, especially, it is critically involved in inflammation and has variable effects on tumors^31,32^. Moreover, MAP3K8 is up-regulated in multiple tumor types and is closely related to tumorigenesis and/or cancer progression^33,34^. The NGF (nerve growth factor) was high correlated with several subtypes of breast cancer, including basal-like breast cancer^35^. PKG2 (Type 2 cGMP-dependent protein kinase) is a major cGMP effector in the gut epithelium, its importance in the regulation of proliferation and differentiation in colon cancer cell lines^35^. Recent evidence suggesting that the PTPN22 might enhance the efficacy of anti-tumour T cell responses^36^.

Moreover, 4 important pathways were isolated from the constructed KEGG-Target network, such as the Jak-STAT signaling pathway, T cell receptor signaling pathway, PI3K-Akt signaling pathway, and cytokine-cytokine receptor interaction. The Western Bolt results demonstrated that GLE was able to significantly mediate signal and cytokine related pathway in tumor treatment process (Fig. 6).

Jak-STAT signaling pathway is critical for the immune system to defend organisms against pathogens and tumor cells, but also to avoid autoimmunity by maintaining immune tolerance^37,38^. In mammals, the Jak family consists of Jak 1, Jak 2, Jak 3 and Tyk 2, and the STAT family including STAT1, −2, −3, −4, −5A, −5B, and −6^39^. In the STAT family, the activated STAT1 leads to target gene transcription^40^. STAT3 is a transcription factor aberrantly activated in many human solid and hematological cancers^41,42^. STAT5A and STAT5B have been described as contributing to breast cancer pathophysiology^39^. STAT6 is the main transducer regulating immune responses to IL-4 and IL-13 signals^43^. Interestingly, the phosphorylated JAKs active STATs phosphorylation, and the phosphorylated STATs dimerize and migrate to the nucleus to regulate target gene^44^. In this work, we found that the Jak3, Stat1, 3, 5, and 6 have had significant difference in GLE group (Fig. 6C,D), and suggested GLE can reduce the Jak3 expression level and then catalyze tyrosine phosphorylation of the STAT family.

T cell receptor signaling pathway is play an important role in immune regulation^45^, and the cascade is tonically repressed by constitutive phosphorylation of the negative regulatory site of Lck at Tyr^505^ ^46^. Active Lck phosphorylates immunoreceptor tyrosine-based activation motifs (ITAMs) in the CD3ζ chain of the TCR complex, leading to recruitment of tyrosine-protein kinase Zap70 and its subsequent phosphorylation by Lck^47^. Therefore, down-regulated p-Lck and p-Zap70 active the T cell receptor signaling pathway.

The PI3K/Akt signaling pathway is an essential pathway, which involve in cell growth, proliferation, cell motility, cell survival, angiogenesis, and cell metabolism, furthermore, it also be important in regulating the innate immune responses and immune modulators^48–51^. The aberrant regulation of the PI3K/Akt/mTOR axis often confers a proliferative advantage to tumor cells and contributes to the development of drug-resistance mechanisms^52,53^.

We further detected 20 serum cytokines in tumor-bearing mice and 7 cytokines were significantly different between GLE treated and model groups, including IFN-γ, IL-1β, IL-4, IL-9, IL-12, RANTES and TNF-α. In these cytokines, IFN-γ is primarily secreted by the natural killer (NK) cells and T cells, which as part of innate immunity and antigen-specific immunity^54^. IL-1β can stimulate the expressions of genes associated with inflammation and autoimmune diseases^55,56^. IL-4 appears to critical function in immunoglobulin (Ig)E synthesis^10^. IL-9 play important roles in a broad spectrum of autoimmune diseases and allergic inflammation^57,58^. In immune responses, IL-12 has been shown to prevent the development of the Th2 immune response in several mice models^59^. RANTES is involved in inflammation and immune response during pathogen infection^60^. TNF-α suppress tumor cell growth by inducing apoptosis in tumor cells^61^. Importantly, the GLPS and triterpene acid were demonstrated to promote the production of cytokines, such as IL-1β, IL-4, IL-6, IL-12, IL-17, RANTES, TNF-α and IFN-γ, and thus to exhibit immuno-modulation and anti-tumor activities^62–67^. These results suggested that GLE regulated serum immune cytokines and implicated the anti-tumor immunostimulatory activity by the cytokine-cytokine receptor interaction pathway.

In conclusion, we established the Hepa1-6-bearing C57 BL/6 mice model to explore the anti-tumor and immunostimulatory activity of GLE and its underlying mechanisms. The results showed that GLE effectively inhibited tumor growth *in vivo* without hepatic/renal toxicity and bone marrow suppression, and might prove more beneficial for immunological function. The microarray, qRT-PCR and Western-bolt reveal that 6 mRNAs might significantly correlate with indicators for GLE treatment. Importantly, the Jak-STAT signaling pathway, T cell receptor signaling pathway, PI3K-Akt signaling pathway, and cytokine-cytokine receptor interaction were high correlated with GLE treatment. Western-blot demonstrated that GLE suppressed growth and proliferation of tumor, which were associated with the inhibition of the Jak-STAT signaling pathway, T cell receptor signaling pathway and PI3K-Akt signaling pathway. The serum cytokine protein chip indicated that regulating serum immune cytokines was implicated in the anti-tumor immunostimulatory activity of GLE treatment.

## Materials and Methods

### Cell culture

The Hepa1-6 cell line was purchased from the Institute of Biochemistry & Cell Biology (Shanghai, China). Then, the cell was cultured in DMEM medium (Gibco, USA), which with 10% fetal bovine serum (FBS) (Gibco, USA) and 100 U/ml penicillin-streptomycin antibiotics. The culture condition is a humidified 5% CO2 incubator (37 °C). Furthermore, the Subculture wasn’t exceed the third generation.

#### Animal model establishment

The 72 C57BL/6 male mice (Four weeks old) were purchased from Shanghai Public Health Center (Experimental animal license Number: SCXK Shanghai 2005-0001), housed and fed in the SPF Animal Laboratory (Animal Laboratory Permit Number: SYXK Shanghai 2005-0008) of Putuo Hospital affiliated with Shanghai University of Traditional Chinese Medicine. The mice were housed in polycarbonate cages with hard wood chips, which in an air-conditioned room (23 ± 2 °C, 55 + 10% R.H.) and keep a 12 h light/dark cycle. This project was approved by the Ethical Committee of Animal Experiments of Shanghai University of Traditional Chinese Medicine, and we confirm that a statement: (i) identifying the institutional and/or licensing committee approving the experiments, including any relevant details; (ii) confirming that all experiments were performed in accordance with relevant guidelines and regulations. In this study, the C57BL/6 mice were weighed and randomly divided into six groups, including control group (n = 12), model group (n = 12), high dose GLE group (n = 12), medium dose GLE group (n = 12), low dose GLE group (n = 12) and (n = 12). Furthermore, the mice of model, GLE and cisplatin group were subcutaneously inoculated in the right flank, respectively, which with 0.1 ml of logarithmic phase Hepa1–6 cells (5 × 10^7^ cells/ml).

#### Drug treatment

According to the conversion of the human clinical dosage and the mouse body surface area, the drug dose of mouse was calculated. Here, the conversion coefficient of human and mouse was 9.1 (the human body weight was defined as 60 kg), and the total dose of a 10 g mouse for one day was 0.023 g (15 g/60 kg × 0.01 kg × 9.1). In the GLE treatment process, low dose was 0.011 g/d, medium dose was 0.023 g/d and high dose was 0.046 g/d, and all GLE was diluted with 0.9% normal saline (0.9% NaCl). From the second day after modeling, the GLE treatment was once a day and 0.2 ml each time for 28 consecutive days. As a control, the cisplatin group mice were treated with 6 mg/kg/d cisplatin (diluted in 0.1 mL 0.9% NaCl saline), which were injected intraperitoneally on the next day after inoculation, and then injected every 6 days. Furthermore, the control group and model group mice were treated with 0.9% NaCl, 0.2 ml per mouse, and once a day for 28 consecutive days.

After a week of inoculation, the mice were weighed every three days. Finally, the mice were sacrificed in the next day of last drug treatment. The transplanted tumor, liver and kidney were immediately separated and weighed. Furthermore, the organ indexes (OI) were calculated, which was defined as OI = organ weight (g)/body weight (g) × 100%.

#### Physiochemical parameters determination

The mice blood samples were collected before kill them, and the automatic blood cell counting apparatus (Mindray, Shenzhen, China) were used to determine the Leukomonocyte (LY) and white blood cells (WBC). Furthermore, the serum was separated from blood samples via centrifugation at 6500 g for 10 min. The activities of serum aspartate aminotransferase (AST), alanine aminotransferase (ALT), total bilirubin (TBIL), blood urea nitrogen (BUN) and creatinine (CRE) were determined by an automatic biochemical analyzer (Sysmex, Japan), respectively.

#### Pathological examination (HE staining)

Mouse liver and kidney was immersed in 4% paraformaldehyde for 4 h, and then transferred to 70% ethanol. Individual lobes of the tissue biopsy were placed in processing cassettes, dehydrated through a serial alcohol gradient, and embedded in paraffin wax blocks. Before immunostaining, the 5-um-thick tissue sections were dewaxed in xylene, and rehydrated by decreasing concentrations of ethanol. After PBS buffer washing, the tissue sections were stained with hematoxylin and eosin (H&E). Finally, the sections were dehydrated through increasing concentrations of ethanol and xylene. The obtained images were measured by pathological image analysis system (Olympus Corporation, Tokyo, Japan).

#### Thymus and bone marrow cell cycle detection

Took the thymus of mice, and subsequently prepared the cell suspension in 2 ml DMEM medium. (i) Cell suspension was centrifuged 1000 rpm for 5 min, washed with 2 ml PBS twice, and filtered with a 300-mesh nylon mesh. After the last washing and centrifugation, 2 ml 75% cold ethanol (4 °C) was slowly added to the fixed cells, and then fixed at −20 °C. (ii) After half an hour, samples were centrifuged (1800 rpm, 10 min) and removed the ethanol, added 2 ml PBS buffer, mixed evenly and washed 2 times, then were centrifuged at 1000 rpm for 5 min, discarding the supernatant and re-suspending the pellet. (iii) The cells were mixed with 0.5 ml PI/RNase for 15 min in the dark, and then the fluorescence activated cell sorting (FACS) was employed to determine the cell cycle distribution. The bone marrow of mouse femur was collected, and the single cell suspension was manufactured with normal saline. The method of cell cycle detecting was as described above.

#### Detection of peripheral blood T cell subsets and NK cells

Peripheral blood samples of mice were collected into heparinized tubes (1 ml). The blood samples (50 μl) were added into the T lymphocyte subset kit (CD3-PE 5 μl, CD4-FITC 2 μl and CD8a-Percp 5 μl) and NK cell kit (CD3-PE 5 μl and NK-1.1-APC 5 μl) (Becton, Dickinson and Company, USA), respectively and stained without light for 20 min. The serum hemolysin (500 μl) was added into the above samples and stained without light for 15 min. Centrifugation at 1000 rpm for 5 min and the supernatant was discarded, 1.5 ml PBS buffer washing and centrifuged at 1000 rpm for 5 min (repeated twice). Then, 400 μl PBS was added to precipitation, and cells were detected by flow cytometry.

#### Transcriptional profiles detection

Three GLE treatment Hepa1-6-bearing C57 BL/6 mice (GLE Group, G) and three Hepa1-6-bearing C57 BL/6 mice (Model Group, M) were used for mRNA profiles detection. Total RNA was extracted using TRIzol reagent (Invitrogen, Grand Island, NY, USA), and the quality evaluation was performed using Agilent 2100 Bioanalyzer (Agilent Technologies, Santa Clara, CA, USA). Furthermore, the Agilent Mouse mRNA Microarrays (4 × 180 K) (Agilent, Santa Clara, CA, USA) were used to detect transcriptional profiles in this experiment. The microarray data were deposited in Gene Expression Omnibus (GEO) database (https://www.ncbi.nlm.nih.gov/geo/).

#### Differentially expressed mRNAs identified

The differentially expressed (DE) mRNAs were identified using R software. In this work, DE mRNAs were defined as fold change ≥ 2.0 and a P-value < 0.05, and the hierarchical clustering was performed to display the DE mRNAs expression pattern among the samples. Using DAVID online program^68^, the enrichment analysis of DE genes were conducted and the significant terms were defined as P value adjusted by False Discovery Rate (FDR). In this study, Gene ontology (GO) and KEGG pathway terms with an FDR-adjusted P-value < 0.05 were retained.

#### Protein-protein interaction (PPI) network

Using BioGrid, IntAct and MINT databases, the DE mRNA-related protein-protein interaction (PPI) network was constructed. In the network, the DE mRNAs were weighted by their expression levels (|log2|), and each gene related pairs were weighted by the degree distributions. Furthermore, the similarities of nodes of network were tested based on their weights, and then, the obtained nodes were used for PPI network rebuilding.

In the PPI network, the topological profiles were calculated based on the ClusterONE algorithm^69^, and the hub clusters were defined as P-value ≤ 0.001, node size ≥6, network density ≥0.05. Subsequently, these hub clusters were used to rebuild a Cluster-related PPI network. In this network, the parameters of Betweenness Centrality (BC), Closeness Centrality (CC), Degree (De) and Topological Coefficient (TC) were calculated respectively, and the kernel genes were defined as BC ≥ Avg (BC), CC ≥ Avg (CC), De ≥ Avg (De) and TC ≥ Avg (TC).

#### KEGG-Target network

Based on the DE mRNAs and related KEGG pathway terms, the KEGG-Target network were constructed. Here, the kernel pathways were defined as BC ≥ Avg (BC), CC ≥ Avg (CC) and De ≥ Avg (De), which were considered as played an important role in GLE treatment process.

#### qRT-PCR validating

Using a Rotor-Gene 6000 Real-time PCR machine (Corbett Life Science, Sydney, Australia), the quantification of kernel mRNAs was performed in GLE treated Hepa1-6-bearing C57 BL/6 mice with SYBR Green PCR Master Mixture (TOYOBO, LTD, Japan). The specificity of PCR products were validated by the melting curves at the end of PCR cycles. In this work, the PCR products were performed in triplicate, and the Ct was considered as the number of cycle requirements for the fluorescent signal to reach the threshold. The levels of mRNA were calculated using 2^ΔCt^, where ΔCt = Ct of internal reference -Ct of the target mRNAs. The significant differences in mRNA expression levels compared using the Student’s t-test and P-value < 0.05. The primer sequences were shown in Table 5, and the levels of mRNAs were normalized to mus-Actb.

**Table 5:** Target- mRNA sequences obtained from the NCBI database.

Gene name	Forward primer(5′- 3′)	Reverse primer(5′- 3′)
mus-Cd3g	ACTGTAGCCCAGACAAATAAAGC	TGCCCAGATTCCATGTGTTTT
mus-Ptpn22	CGACAAACCAGCATAGCAAA	GGACACGACCCAAACAACAT
mus-Prkg2	AAGGACGCGGAACTTCAGG	GAGGCACTTTGTCGGGAGAG
mus-Map3k8	ATGGAGTACATGAGCACTGGA	GGCTCTTCACTTGCATAAAGGTT
mus-Ngfr	TGCCGATGCTCCTATGGCTA	CTGGGCACTCTTCACACACTG
mus-Gnas	GTCATCTGCACCTTTGCACTT	GGCTGCCTAAGAGTTAGCGTC
mus-Actb	GGCTCCTAGCACCATGAAGA	AGCTCAGTAACAGTCCGCC

#### Western-blot validating

For western blot, the tissues were lysed in RIPA buffer, which containing 50 mmol/L Tris/HCl (pH 8.0), 150 mmol/L NaCl, 1% Nonidet-P40, 1% sodium deoxycholate, 0.1% SDS, 0.1 mmol/L DTT, 0.05 mmol/L PMSF, 0.002 mg/ml aprotinin, 0.002 mg/ml leupeptin, and 1 mmol/L NaVO3. The BCA protein assay was used to determine the protein concentration of each supernatant. Equal amounts of protein were loaded and separated by 12% SDS-PAGE, and then, the proteins were transferred onto polyvinylidene difluoride membranes. The membranes were incubated overnight with appropriate primary antibodies at 4 °C, which including CD3G, GNAS, MAP3K8, NGFR, PRKG2, PTPN22, JAK 1, JAK 2, JAK 3, p-Stat 1, p-Stat 3, p-Stat 5, p-Stat 6, IKKα, IKKβ, p-IKKα/β, NF-κB, p-NF-κB, IκBα, p-IκBα, CD3ε (CD3-12), p-Lck, p-PLCγ1 (Tyr783), p-SLP-76, p-Src, p-Zap-70, PI3K(p85), Akt, p-Akt, mTOR, p-mTOR. Furthermore, the membranes were further incubated with HRP-conjugated secondary antibodies (anti-rabbit or mouse immunoglobulin G) for 1 h at 25 °C. The immunoreactivity was detected by enhanced chemiluminescence (ECL) (Bio-Rad, USA), and β-actin was conducted as a loading control. The Image Lab™ software was performed for quantitative analysis, and all reagents were purchased from Cell Signaling Technology of USA.

#### Mouse cytokines quantitative measurement

The arterial blood was collected and centrifuged 3500 rpm for 10 min, and the upper serum was collected. Furthermore, the same group’s serums were mixed and centrifuged 10000 rpm for 10 min, repeated 2 times, and the obtained upper serum was moved into the new centrifuge tube. In accordance with the Quantibody® Mouse Cytokine Array 1 (Raybiotech company, USA) supporting the process of hybridization standard chip detection, the process was as follows: (1) serum dilution, (2) chip closed, (3) chip hybridization, (4) chip scan, (5) image obtained. The original data were read using GenePix Pro 6.0 software, including fluorescent signals and background. The fluorescence signal FI (F532, Median-B532Median) was used to analyze the fluorescence signal, which reflects the expression level of the cytokine. A total of 20 cytokines were tested and four replicates per antibody on the chip were conducted. Finally, the average values of the repeated four replications were calculated as each signal factor value, then the positive control signal values between samples were normalized, and finally the normalized data were used to quantify concentration. The array data were deposited in Protein Microarray Database (PMD) (http://proteinmicroarray.cn/).

### Statistical analysis

The statistical analysis was performed using *SPSS* 21.0 software package, and all quantitative data were presented with mean ± *SD* at three independent experiments. Comparisons between two groups were performed using *T* test analysis, and between multiple groups using *ANOVA* analysis. The *P* value < 0.05 was considered statistically significant.

## Electronic supplementary material

Supplementary information

## Data Availability

All data generated or analyzed during this study are included in this published article (and its Supplementary Information files).
