# Supplementary material for: The effect of Ganoderma lucidum extract on immunological function and identify its anti-tumor immunostimulatory activity based on the biological network
Source: Sci Rep. 2018 Aug 23;8:12680. doi: 10.1038/s41598-018-30881-0 (PMC6107651; doi:10.1038/s41598-018-30881-0)

The effect of Ganoderma lucidum extract on immunological function and identify its anti-tumor immunostimulatory activity based on the biological network

Ruolin Zhao [1,2], Qilong Chen [1,2#], Yu-min He [1#]

[1] Shanghai University of Traditional Chinese Medicine, Shanghai 201203

[2] Research Center for TCM Complexity System, Shanghai University of TCM, Shanghai 201203, China

* These authors are contributing for this paper.

Correspondence to: Qilong Chen, email: cqlw1975@126.com, and Yu-min He, **email:** heyumin109109@sina.com , Address: No.1200 Cailun Road, Shanghai, 201203, China.

Supplementary data 1: The 6 kernel mRNAs related encoding proteins were validated using Western-blot, which including GNAS, PTPN22, CD3G, PRKG2, MAP3K8 and NGFR. The β-actin was used as a loading control.

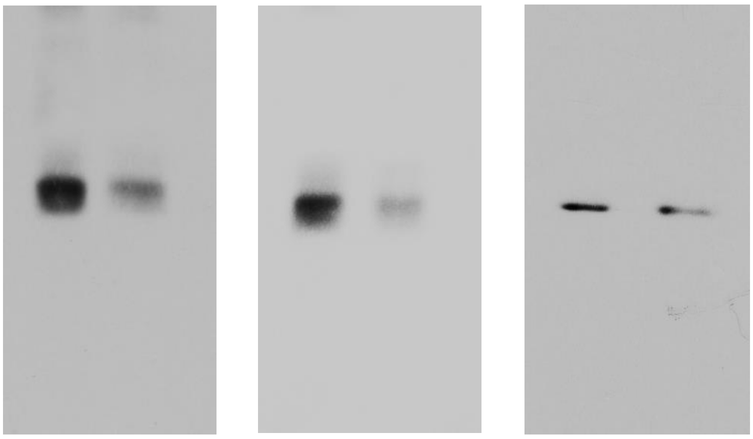

GNAS PTPN22 CD3G

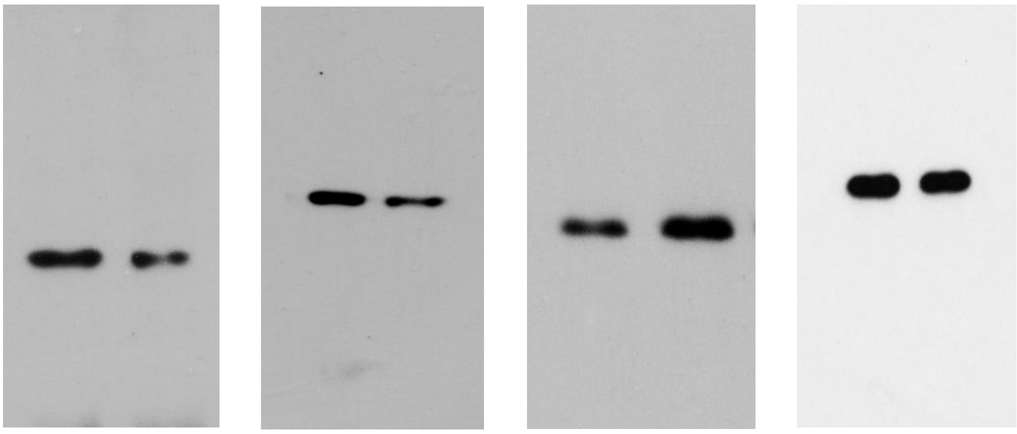

PRKG2 MAP3K8 NGFR actin

Supplementary data 2:

(A) The Jak/Stat signaling pathway related proteins were validated using Western-blot, and the expression levels of JAK3, p-Stat1, p-Stat3, p-Stat5 and p-Stat6 were significantly inhibited by GLE in vivo; (B) The T cell receptor signaling pathway related proteins were validated using Western-blot, and the expression levels of p-Lck and p-Zap-70 were down-regulated; (C) The PI3K/Akt/mTOR signaling pathway related proteins were validated using Western-blot, the expression levels of p-Akt and p-mTOR were down-regulated, and PI3K was up-regulated. The β-actin was used as a loading control.

(A)

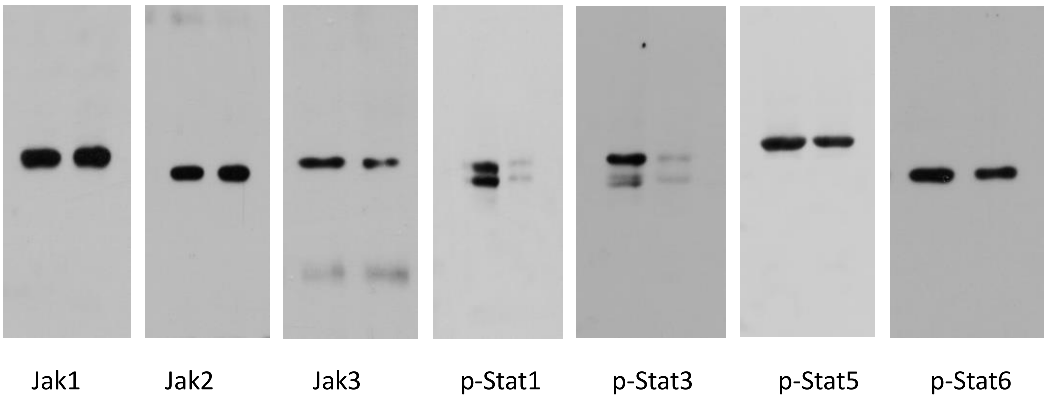

(B)

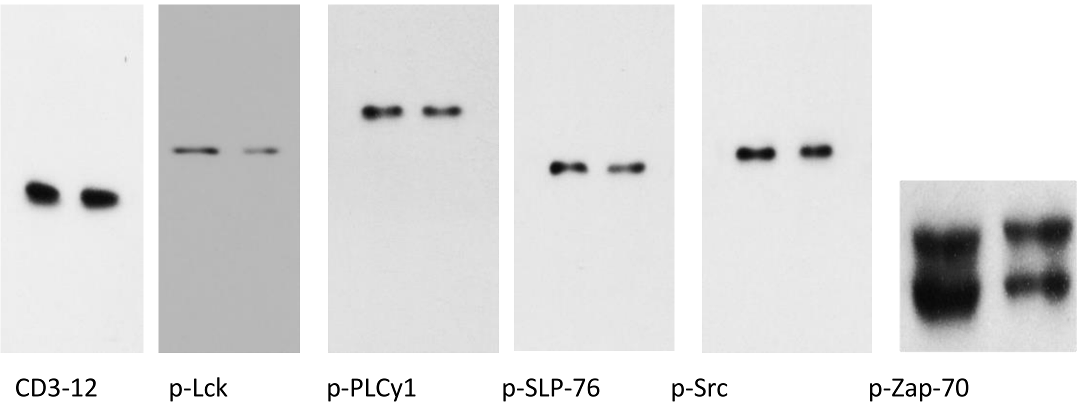

(C)

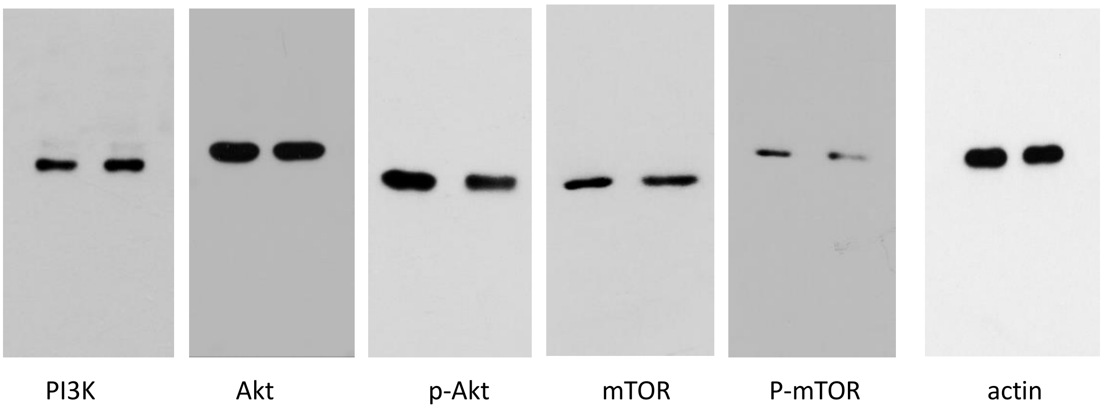

Supplement: Supplementary file 1 — Supplementary information [file 41598_2018_30881_MOESM1_ESM.pdf]
